# Thermodynamic Assessment of CaO-Al_2_O_3_-Fe_2_O_3_ System

**DOI:** 10.3390/ma19143136

**Published:** 2026-07-21

**Authors:** Wenqing Zhao, Lideng Ye, Junfeng Wu, Hong Chen, Ligang Zhang, Libin Liu

**Affiliations:** School of Materials Science and Engineering, Central South University, Changsha 410083, China; zhaowenqing2002@163.com (W.Z.); yelideng0615@163.com (L.Y.); 243112151@csu.edu.cn (J.W.); chenh200307@163.com (H.C.)

**Keywords:** CaO-Al_2_O_3_-Fe_2_O_3_ system, thermodynamic modeling, phase diagram, CALPHAD

## Abstract

The CaO-Al_2_O_3_-Fe_2_O_3_ system is widely encountered in cement production, iron ore sintering, metallurgical slags, and refractory materials. A thermodynamic assessment of the CaO-Fe_2_O_3_ and CaO-Al_2_O_3_-Fe_2_O_3_ systems was carried out in this study based on the CALculation of PHAse Diagrams (CALPHAD) method. The liquid was modeled using the ionic two-sublattice model, expressed as (Ca^+2^, Al^+3^, Fe^+2^) _P_ (O^−2^, AlO_1.5_, FeO_1.5_, Va, O) _Q_. The Compound Energy Formalism (CEF) was adopted to describe compounds and solid solutions. A self-consistent thermodynamic assessment of the CaO-Fe_2_O_3_ and CaO-Al_2_O_3_-Fe_2_O_3_ systems was achieved, enabling accurate reproduction of phase equilibrium and thermodynamic data. The obtained thermodynamic description provides a useful foundation for the design, optimization, and processing of refractory materials.

## 1. Introduction

The CaO-Al_2_O_3_-Fe_2_O_3_ ternary system is of significant fundamental and practical importance in the fields of metallurgy, construction materials, and refractory engineering, and represents a key subsystem in high-temperature oxide thermodynamics. In steelmaking processes, this system is widely present in slags from basic oxygen furnaces, electric arc furnaces, and secondary refining operations. Its composition strongly influences slag basicity, melting temperature, viscosity, and the kinetics of desulfurization and dephosphorization reactions, thereby governing the thermodynamic efficiency and operational stability of steel production [1,2]. In addition, phases related to the CaO-Al_2_O_3_-Fe_2_O_3_ system exhibit excellent high-temperature stability and strong resistance to slag corrosion, making them suitable for the design and optimization of ladle castables and advanced refractory materials [3]. Therefore, a detailed understanding of the phase equilibria and thermodynamic behavior of this system is essential for elucidating reaction mechanisms in high-temperature oxide systems and guiding the design of industrial materials [4]. In cement clinker production, calcium alumino-ferrite phases typically exist as solid solutions; the formation and transformation of these phases significantly influence the mineralogical composition of the clinker and, consequently, its strength development. Therefore, a comprehensive understanding of their phase equilibrium characteristics is crucial for controlling phase changes during material synthesis and application [5]. To obtain phase equilibrium data efficiently, appropriate thermodynamic databases and specialized calculation software should be employed [6].

The CALPHAD method is a powerful computational thermodynamic approach used to model and predict phase equilibria and thermodynamic properties in multicomponent systems [7]. It is widely applied in materials science, metallurgy, ceramics, and geosciences. The core idea of CALPHAD is to describe the Gibbs free energy of each phase in a system using physically based thermodynamic models and optimized parameters derived from critically assessed experimental data and, when available, first-principles calculations. Once reliable Gibbs energy descriptions are established for all relevant phases, equilibrium states are determined by minimizing the total Gibbs free energy of the system under given conditions of temperature, pressure, and composition. By combining experimental information with thermodynamic modeling, CALPHAD provides a self-consistent framework that allows extrapolation from simple binary systems to complex multicomponent systems [8]. This makes it a key tool for constructing phase diagrams, calculating phase stability, and supporting materials design and process optimization [9].

Based on the available experimental information, the CaO-Fe_2_O_3_ and CaO-Al_2_O_3_-Fe_2_O_3_ systems were thermodynamically evaluated using the CALPHAD approach. The liquid phase was described by the ionic two-sublattice model, while compound and solid-solution phases were represented using the sublattice CEF model. A self-consistent thermodynamic database was thereby established, providing an accurate description of the phase equilibria in the CaO-Al_2_O_3_-Fe_2_O_3_ system. It should be noted that systems containing Fe_2_O_3_ are not strictly binary because Fe^+3^ and Fe^+2^ coexist under equilibrium conditions. Owing to the Fe^+3^/Fe^+2^ redox equilibrium at elevated temperatures, Fe^+2^ species are present in the liquid phase, and spinel phases containing Fe^+2^ may become stable in the CaO-Fe_2_O_3_ and Al_2_O_3_-Fe_2_O_3_ systems. Nevertheless, under a fixed oxygen partial pressure, when the temperature is above the stability range of the spinel phase, the corresponding section resembles a true binary phase diagram. Therefore, the present assessment focuses on phase equilibria under fixed oxygen partial pressure, and the calculated phase diagrams involving Fe_2_O_3_ should be regarded as constant oxygen partial pressure sections. This clarification defines the scope of the present thermodynamic modeling and applies to the complete CaO-Al_2_O_3_-Fe_2_O_3_ system.

## 2. Review of Literature Data

### 2.1. CaO-Al_2_O_3_ System

The CaO-Al_2_O_3_ binary system contains four intermediate compounds, namely 3CaO·Al_2_O_3_ (C_3_A), CaO·Al_2_O_3_ (CA), CaO·2Al_2_O_3_ (CA_2_), and CaO·6Al_2_O_3_ (CA_6_).

Experimental investigations by several researchers [10,11,12,13] demonstrated that C_12_A_7_ corresponds to the hydrated compound Ca_12_Al_1_4O_32_(OH)_2_ and does not belong to the stable anhydrous CaO-Al_2_O_3_ binary system. Consequently, C_12_A_7_ has generally been excluded from recent thermodynamic assessments of the CaO-Al_2_O_3_ system.

Experimental studies of the phase equilibria in the CaO-Al_2_O_3_ binary system were initiated at an early stage. In 1955, Wisnyi et al. [14] reported the liquidus temperatures in the Al_2_O_3_-rich region and the invariant reaction temperatures based on Petrographic Microscopy Analysis, Thermal Analysis, and X-ray Diffraction (XRD). Subsequently, Nurse et al. [11] and Rolin [15] independently measured the invariant reaction temperatures and liquidus data. In 1983, Nityanand and Fine [16] supplemented the liquidus measurements with Heating-stage Microscope under an argon atmosphere. Later, Jerebtsov and Mikhailov [12] systematically investigated the invariant equilibria in the CaO-Al_2_O_3_ system by Differential Thermal Analysis (DTA) and reported the corresponding temperatures, compositions, and liquidus boundaries.

Bonnickson [17] determined the high-temperature enthalpy increments of C_3_A, CA, and CA_2_ by Thermal Analysis and XRD. King [18] measured the low-temperature heat capacities of C_3_A, CA, and CA_2_ using Adiabatic calorimetry. In addition, numerous calorimetric studies [19,20,21,22,23] have reported the enthalpies of formation of solid phases in the CaO-Al_2_O_3_ system. Kumar and Kay [24] as well as Allibert et al. [25] measured the activity of CaO in several two-phase regions over the temperature range of 900–1500 K. Allibert et al. [25] reported activity measurements for CaO and Al_2_O_3_ at 2060 K obtained by Knudsen effusion mass spectrometry (KEMS).

**Figure 1:**
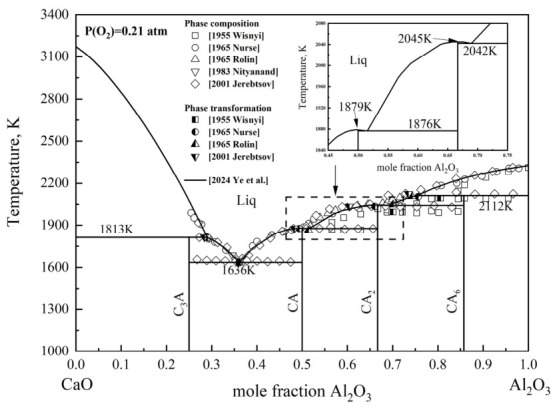
Calculated CaO-Al_2_O_3_ phase diagram according to the thermodynamic models and parameters of Ye et al. [29], compared with the experimental data [11,12,14,15,16].

Hallstedt [26] performed a thermodynamic assessment of the liquid phase using the ionic two-sublattice model (Ca^+2^, Al^+3^)(O^−2^). Eriksson et al. [10] employed the Modified Quasi-chemical Model (MQM) to describe the liquid phase. However, this model is incompatible with the ionic two-sublattice formalism adopted in the present work, and therefore its parameters cannot be directly used. Mao et al. [27] described the liquid phase using the model (Ca^+2^, Al^+3^) _P_ (AlO_2_^−1^, O^−2^) _Q_. Although improvements were introduced, the assessment still exhibited several shortcomings, including discrepancies between calculated and experimental enthalpies of formation of compounds, unrealistic decomposition behavior of compounds at low temperatures, and insufficient agreement with invariant reaction data. More recently, Guo et al. [28] adopted the liquid model and thermodynamic parameters proposed by Hallstedt [26] to calculate the phase diagram, achieving satisfactory agreement with available experimental phase-equilibrium data. Nevertheless, the estimated enthalpies of formation for CA_2_ and CA_6_ in these assessments deviate significantly from experimentally measured values, which affects the accuracy of phase-equilibrium predictions in the Al_2_O_3_-rich region, particularly at low temperatures. To address this issue, Ye et al. [29] critically reassessed the experimental enthalpy data of intermediate compounds, including CA_2_ and CA_6_. The CaO-Al_2_O_3_ system was re-optimized using the ionic two-sublattice model (Ca^+2^, Al^+3^) _P_ (AlO_1.5_, O^−2^, O, Va) _Q_. The resulting thermodynamic parameters were shown to reproduce both the phase-equilibrium relationships and thermochemical data of the system with high accuracy. Accordingly, the thermodynamic description developed by Ye et al. [29] was employed in the present assessment. The resulting calculated phase diagram of the CaO-Al_2_O_3_ binary system is presented in Figure 1.

### 2.2. CaO-Fe_2_O_3_ System

The CaO-Fe_2_O_3_ binary system contains three intermediate compounds: Ca_2_Fe_2_O_5_ (C_2_F), CaFe_2_O_4_ (CF), and CaFe_4_O_7_ (CF_2_).

Phillips and Muan [30] established the phase boundaries of the CaO-Fe_2_O_3_ system through quenching experiments combined with XRD and optical microscopy analyses. Scheel [31] measured the liquidus of the CaO-Fe_2_O_3_ system under an oxygen partial pressure of P(O_2_) = 0.21 atm. Timucin and Morris [32] further determined the liquidus boundaries of the system at P(O_2_) = 1 atm. Cheng et al. [33] investigated the liquidus and phase equilibrium relationships between 1393 and 2013 K under various oxygen partial pressures using a quenching technique combined with Electron Microprobe Analysis (EMPA).

For the CF phase, King [34] measured the low-temperature heat capacity over the temperature range of 52.3–298.16 K using adiabatic calorimetry and derived an entropy of 145.2 ± 0.8 J/mol·K at 298.15 K. Rajagopalan et al. [35] measured the heat capacity of CF between 297 and 1091 K using the laser-flash technique and reported values significantly higher than the sum of the heat capacities of the constituent oxides. The Gibbs free energy of formation of CF from the component oxides was measured by Rezukhina and Bagin’ska [36], Jacob et al. [37], and Forsberg et al. [38].

The low-temperature heat capacity of C_2_F was measured by King [34] from 53.3 to 298.16 K by adiabatic calorimetry and calculated an entropy of 188.7 ± 1.3 J/mol·K at 298.15 K. The heat capacity of C_2_F was investigated by Rajagopalan et al. [35] over the temperature range of 296–1090 K, and the measured values showed good agreement with those calculated from the additive heat capacities of the constituent oxides. Bonnickson [39] measured the enthalpy increment of C_2_F between 376.5 and 1838 K. In addition, several researchers [36,37,38] have reported the Gibbs free energy of the reaction CaO + CF = C_2_F.

Thermodynamic information for the CF_2_ phase remains rather limited. The stability region of CF_2_ was reported by Phillips and Muan [30] to span 1428–1499 K in air and 1445–1501 K under P(O_2_) = 1 atm. Edström [40] investigated the stability of CF_2_ by means of sintering, quenching, and XRD, and reported a stability range of 1393–1501 K at P(O_2_) = 1 atm. Batti [41] reported that CF_2_ is stable in air over the temperature range 1403–1503 K.

Hillert et al. [42] were the first to use the ionic two-sublattice model (Ca^+2^, Fe^+2^, Fe^+3^) _P_ (O^−2^, Va) _Q_ to describe the liquid phase in the CaO-Fe_2_O_3_ system. Selleby et al. [43] subsequently improved the liquid description by adopting the model (Ca^+2^, Fe^+2^) _P_ (O^−2^, Va, FeO_1.5_) _Q_ and re-optimized the system based on the work of Hillert et al. [42]. Although both assessments produced a reasonable set of thermodynamic parameters, several newly available experimental data sets [44,45] were not considered, leading to discrepancies between calculated and experimental results. In 2016, Hidayat et al. [46] critically evaluated all available experimental data for the CaO-Fe_2_O_3_ system and obtained a self-consistent thermodynamic description using the MQM for the liquid phase. More recently, Nekhoroshev et al. [47] optimized the system using the MQM by incorporating the experimental data reported by Cheng et al. [33]. However, a thermodynamic reassessment based on the ionic two-sublattice model that incorporates these newly available experimental data has not yet been reported. Therefore, the present work re-evaluates and optimizes the thermodynamic parameters of the CaO-Fe_2_O_3_ system using the ionic two-sublattice model (Ca^+2^, Fe^+2^) _P_ (O^−2^, FeO_1.5_, Va) _Q_.

### 2.3. Al_2_O_3_-Fe_2_O_3_ System

The Al_2_O_3_-Fe_2_O_3_ system contains only one intermediate compound, Fe_2_Al_2_O_6_.

Muan and Gee [48] determined the phase-equilibrium relationships in the Al_2_O_3_-Fe_2_O_3_ system over the temperature range of 1358–1998 K under air and 1 atm oxygen atmospheres using the quenching technique. The approximate compositions of the liquid phase were established. In addition, a number of researchers [49,50,51,52,53,54,55,56] studied the phase equilibria of this system over a range of oxygen partial pressures. Owing to the presence of Fe^+2^ in the liquid phase, the Al_2_O_3_-Fe_2_O_3_ system is not a true binary system. At high temperatures, Fe_2_O_3_ partially dissociates into FeO and O_2_, resulting in the formation of a spinel phase. However, under a fixed oxygen partial pressure, the corresponding section resembles a true binary phase diagram in the temperature range where the spinel phase is no longer stable. Accordingly, the phase-equilibrium data reported in Refs. [49,50,51,52,53,54,55,56] under constant oxygen partial pressures are commonly presented and discussed as approximate binary phase diagrams of the Al_2_O_3_-Fe_2_O_3_ system.

Several studies [48,49,54,56] showed that the high-temperature stability limit of Fe_2_Al_2_O_6_ corresponds to its decomposition into spinel and corundum. Muan and Gee [48] investigated the Fe_2_Al_2_O_6_ phase using quenching, XRD, and chemical analysis, and reported that the decomposition temperature increases from 1683 K to 1763 K as the oxygen partial pressure increases from 0.21 atm to 1 atm. Atlas and Sumida [49] synthesized the Fe_2_Al_2_O_6_ compound by sintering mechanically mixed coprecipitated powders and characterized quenched samples using XRD and optical pyrometry. Their results indicated that the low-temperature stability limit of the Fe_2_Al_2_O_6_ compound was approximately 1593 K. Feenstra et al. [55] obtained the Fe_2_Al_2_O_6_ phase through prolonged reaction of γ-Al_2_O_3_ and α-Fe_2_O_3_ in air and characterized the resulting samples using EPMA and XRD. In contrast, Rhamdhani et al. [56] observed the coexistence of the Fe_2_Al_2_O_6_ compound and corundum at 1573 K. Within the Al_2_O_3_-Fe_2_O_3_ system in air, the phase boundaries of the spinel region were determined in Refs. [48,56] and the reported results were in excellent agreement.

**Figure 2:**
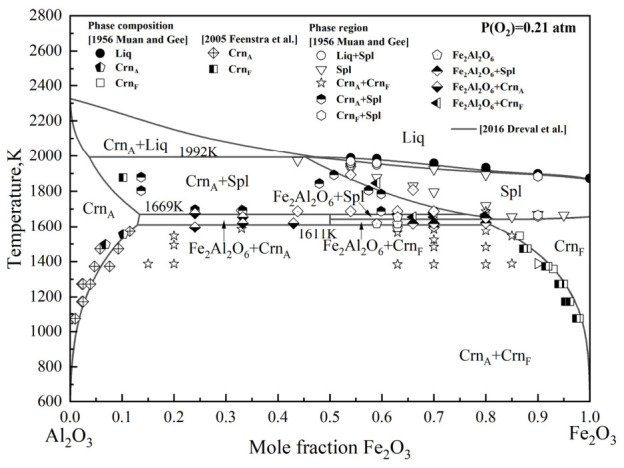
Calculated Al_2_O_3_-Fe_2_O_3_ phase diagram according to the thermodynamic models and parameters of Dreval et al. [59], compared with the experimental data [48,55].

Shishin et al. [57] performed a thermodynamic assessment and optimization of the Al-Fe-O system, in which the MQM was used to describe the liquid phase. For the Al_2_O_3_-Fe_2_O_3_ system, Gao et al. [58] described the liquid phase using the ionic two-sublattice model (Al^+3^, Fe^+2^) _P_ (AlO_2_^−1^, FeO_1.5_, O^−2^, Va) _Q_. Dreval et al. [59] used (Al^+3^, Fe^+2^) _P_ (O^−2^, AlO_1.5_, FeO_1.5_, Va) _Q_. To ensure the consistency of the ionic two-sublattice model in each system, this article adopts the liquid model by Dreval et al. [59]. The calculated Al_2_O_3_-Fe_2_O_3_ phase diagram is presented in Figure 2.

### 2.4. CaO-Al_2_O_3_-Fe_2_O_3_ System

The phase equilibrium behavior of the CaO-Al_2_O_3_-Fe_2_O_3_ system at 1418, 1443, 1573, and 1603 K in air was investigated by Dayal and Glasser [60] using a combination of quenching, optical microscopy, and XRD. Based on their results, isothermal sections and a liquidus projection of the ternary system were established. They also identified a ternary compound, Ca(Al,Fe)_6_O_10_, denoted as C(A,F)_3_. According to Lister and Glasser [61], the C(A,F)_3_ phase remains thermodynamically stable in O_2_ from 973 to 1743 K. Cheng et al. [62] reported that this phase was synthesized by Malysheva et al. and its homogeneity range was determined. In a later study, Chen et al. [63] evaluated the stability of C(A,F)_3_ in both air and high-purity CO_2_ atmospheres over the temperature range of 1423–1523 K. Their results showed that the phase remained stable over a relatively wide compositional range of 10.9–30.4 mol.% Al_2_O_3_, although its stability decreased with decreasing oxygen partial pressure. Hassaan et al. [64] measured the solubility limit of Fe_2_O_3_ in the C_3_A phase and reported a maximum solubility of 4 wt.%. Using high-temperature equilibration experiments followed by quenching and electron probe microanalysis (EPMA), Cheng et al. [62] examined the phase equilibria of the FeO-Fe_2_O_3_-CaO-Al_2_O_3_ system in air. The results indicated that the C(A,F)_3_ phase melted incongruently and possessed a primary crystallization field within the temperature range of 1462–1686 K. The Al_2_O_3_ content in the C(A,F)_3_ phase increased with decreasing temperature. Cheng et al. [62] also reported another ternary compound, C_2_F_3_A, which remained stable up to 1498 K and melted incongruently. Based on these phase-equilibrium data, a liquidus projection of the CaO-Al_2_O_3_-Fe_2_O_3_ system centered on the C(A,F)_3_ phase field was established. The phase-equilibrium data reported by Chen et al. [63] at 1523 K in air suggested a narrower solid-solution range for the C(A,F)_3_ phase than that reported by Cheng et al. [62].

Thermodynamic assessments of the CaO-Al_2_O_3_-Fe_2_O_3_ system remain relatively limited. To date, Gao et al. [65] have been the only researchers who have described the liquid phase using the ionic two-sublattice model (Al^+3^, Ca^+2^, Fe^+2^) _P_ (AlO_2_^−1^, FeO_1.5_, O^−2^, Va) _Q_. However, no thermodynamic assessment employing an AlO_1.5_-based liquid-phase description for Al_2_O_3_ has yet been reported for this system. Therefore, in the present work, AlO_1.5_ is introduced into the liquid-phase model, and the ionic two-sublattice model (Al^+3^, Ca^+2^, Fe^+2^) _P_ (AlO_1.5_, FeO_1.5_, O^−2^, O, Va) _Q_ is adopted to describe the liquid phase of the CaO-Al_2_O_3_-Fe_2_O_3_ system. Combined with the experimental phase-equilibrium data reported by Cheng et al. [62], this work re-evaluates and optimizes the thermodynamic parameters of the liquid phase and ternary compounds.

## 3. Thermodynamic Modeling

The crystal structures and thermodynamic models of all stable phases in the CaO-Al_2_O_3_-Fe_2_O_3_ system are listed in Table 1. A detailed description of the thermodynamic models employed in the present assessment is provided in the following sections.

### 3.1. Unary Components

In the CaO-Al_2_O_3_-Fe_2_O_3_ system, CaO is modeled as a stoichiometric phase. The Gibbs energy of the CaO phase is given by:

(1) G  0− HSER = a + bT + cTlnT + dT2 + eT−1 + fT3,

where *H^SER^* represents the standard molar enthalpy of the pure elements (Ca and O) in their Standard Element Reference (SER) states at 298.15 K and 101,325 Pa (J·mol^−1^); *T* is the absolute temperature (K); and *a–f* are adjustable parameters to be optimized. The thermodynamic parameters of the pure CaO oxide end-member used in the present work were adopted from the assessment reported by Hallstedt [26].

Al_2_O_3_ and Fe_2_O_3_ share the same crystal structure, forming the solid solution: corundum [48,49,51,52,53,54,55]. The corundum phase, formulated as (Al^+3^,Fe^+3^)_2_(O^−2^)_3_, is modeled thermodynamically using the sublattice CEF model proposed by Dreval et al. [59].

(2) Gmcorundum=yAl+3yFe+3(LAl+3,Fe+3:O−2corundum 0+LAl+3,Fe+3:O−2corundum 1(yAl+3−yFe+3))

where *y* is the site fraction of each species in their own sublattices in the corundum phase, *L* are the interaction parameters that utilize the Redlich–Kister polynomials in the corundum phase.

### 3.2. Liquid

The liquid phase of the CaO-Al_2_O_3_-Fe_2_O_3_ system is modeled using the ionic two-sublattice liquid model, (Al^+3^, Fe^+2^, Ca^+2^) _P_ (O^−2^, AlO_1.5_, FeO_1.5_, Va, O) _Q_. Here, P and Q represent the site numbers of the cationic and anionic sublattices, respectively. To satisfy the electroneutrality condition, the stoichiometric coefficients P and Q are composition dependent. The Gibbs energy of the liquid phase is given by:

(3) Gmliquid − HSER = yCa+2yO−2GCa+2:O−2liquid + yFe+2yO−2GFe+2:O−2liquid + yAl+3yO−2GAl+3:O−2liquid + Q(yCa+2yVaGCa+2:Valiquid + yAl+3yVaGAl+3:Valiquid + yFe+2yVaGFe+2:Valiquid + yAlO1.5GAlO1.5liquid + yFeO1.5GFeO1.5liquid) + PRT(yCa+2lnyCa+2 + yFe+2lnyFe+2 + yAl+3lnyAl+3) + QRT(yAlO1.5lnyAlO1.5 + yFeO1.5lnyFeO1.5 + yO−2lnyO−2 + yValnyVa + yOlnyO) + Gmliquid E,

*y* is the site fraction of each species in their own sublattices in the liquid phase, $G^{\mathrm{liquid}}$ is the Gibbs energy of end-member and *R* is the gas constant (*R* = 8.314 J/(mol·K)). Gmliquid E is the excess Gibbs energy and is defined by:

(4) Gmliquid = yAl+3 EyO−2yVaLAl+3:O−2,Valiquid + yAl+3yVayAlO1.5LAl+3:Va,AlO1.5liquid + yFe+2yO−2yFeO1.5LFe+2:O−2,FeO1.5liquid + yFe+2yO−2yVaLFe+2:O−2,Valiquid + yFe+2yFeO1.5yVaLFe+2:FeO1.5,Valiquid + yCa+2yO−2yVaLCa+2:O−2,Valiquid + yCa+2yFe+2yO−2LCa+2,Fe+2:O−2liquid + yCa+2yO−2yFeO1.5LCa+2:O−2,FeO1.5liquid + yCa+2yFe+2yO−2yFeO1.5LCa+2,Fe+2:O−2,FeO1.5liquid + yAlO1.5yOLAlO1.5,Oliquid + yCa+2yO−2yAlO1.5LCa+2:O−2,AlO1.5liquid + yAlO1.5yFeO1.5LAlO1.5,FeO1.5liquid + yAl+3yFe+2yVaLAl+3,Fe+2:ValiquidyFe+2yVayAlO1.5LFe+2:Va,AlO1.5liquid + yFe+2yO−2yAlO1.5LFe+2:O−2,AlO1.5liquid + yFe+2yO−2yAlO1.5yFeO1.5LFe+2:O−2,AlO1.5,FeO1.5liquid+ yCa+2yO−2yAlO1.5yFeO1.5LCa+2:O−2,AlO1.5,FeO1.5liquid

where *L* are the interaction parameters that utilize the Redlich–Kister polynomials in the liquid phase.

### 3.3. Intermediate Compounds

The intermediate compounds in the CaO-Al_2_O_3_ system, including C_3_A, CA, CA_2_, and CA_6_, were treated as stoichiometric compounds. The Gibbs energy of these phases is expressed as follows:

(5) GmCxAy = x·GCaOsolid 0 + y·GAl2O3solid 0 + a + bT + cTln(T),

where *x* and *y* denote the molar ratios of CaO and Al_2_O_3_ in each compound, respectively. GCaOsolid 0 and GAl2O3solid 0 represent the Gibbs energies of the pure solid end-members CaO and Al_2_O_3_, respectively. The parameters a, b, and c are adjustable parameters to be optimized in the present work, which are related to the enthalpy and entropy of formation of the compounds.

The CaO-Fe_2_O_3_ system contains three intermediate compounds that require thermodynamic modeling. The Gibbs energy expressions for CF and C_2_F are given as follows:

(6) G 0 − HSER = a + bT + cTlnT + dT2 + eT−1,

where $H^{\mathrm{SER}}\;$ represents the standard molar enthalpy of the pure elements (Ca, Fe, and O) in their Standard Element Reference (SER) states at 298.15 K and 101,325 Pa (J·mol^−1^); T is the absolute temperature in Kelvin (K); and *a–e* are adjustable parameters to be optimized.

The Gibbs energy of CF_2_ is expressed as follows:

(7) GmCxFy = x·GCaOsolid 0 + y·GFe2O3solid 0 + a + bT,

where *x* and *y* represent the molar ratios of CaO and Fe_2_O_3_ in each chemical formula, respectively. GCaOsolid 0  and GFe2O3solid 0 denote the Gibbs energies of the pure solid end-members CaO and Fe_2_O_3_, respectively. The parameters *a* and *b* are adjustable parameters to be optimized, which are related to the enthalpy and entropy of formation of the compound.

The Gibbs free energy of the intermediate compound Fe_2_Al_2_O_6_ in the Al_2_O_3_-Fe_2_O_3_ system is expressed as:

(8) $$G_{m}^{\mathrm{Fe}2\mathrm{Al}2O6}\;-{\;H}^{\mathrm{SER}}\;=\;a\;+\;\mathrm{bT}\;+\;\mathrm{cTlnT}\;+\;dT^{2}\;+\;eT^{-1}$$

In the Al_2_O_3_-Fe_2_O_3_ system, Dreval et al. [59] modeled the spinel phase as (Al^+3^, Fe^+2^, Fe^+3^${)}_{1}^{T}$(Al^+3^, Fe^+2^, Fe^+3^, Va${)}_{2}^{O}$(Va)_2_(O^−2^)_4_. When CaO is present in the system, Ca^+2^ enters the second sublattice [42,59]. In the CaO-Fe_2_O_3_ system [42,43], the spinel phase is described as (Fe^+2^, Fe^+3^${)}_{1}^{T}$(Ca^+2^, Fe^+2^, Fe^+3^, Va${)}_{2}^{O}$(Fe^+2^, Va)_2_(O^−2^)_4_. In summary, the sublattice CEF model of the spinel phase can be expressed as (Al^+3^, Fe^+2^, Fe^+3^${)}_{1}^{T}$(Al^+3^, Ca^+2^, Fe^+2^, Fe^+3^, Va${)}_{2}^{O}$(Fe^+2^, Va)_2_(O^−2^)_4_. The Gibbs energy expression of the spinel phase is given as follows:

(9) Gmspinel − HSER=yAl+3TyAl+3OyVayO−2GAl+3:Al+3:Va:O−2spinel+ yAl+3TyCa+2OyVayO−2GAl+3:Ca+2:Va:O−2spinel + yAl+3TyFe+2OyVayO−2GAl+3:Fe+2:Va:O−2spinel + yFe+2TyAl+3OyVayO−2GFe+2:Al+3:Va:O−2spinel + yFe+2TyFe+2OyVayO−2GFe+2:Fe+2:Va:O−2 spinel+ yAl+3TyFe+3OyVayO−2GAl+3:Fe+3:Va:O−2spinel + yFe+2TyFe+3OyVayO−2GFe+2:Fe+3:Va:O−2spinel + yFe+3TyAl+3OyVayO−2GFe+3:Al+3:Va:O−2spinel + yFe+3TyFe+2OyVayO−2GFe+3:Fe+2:Va:O−2spinel + yFe+3TyFe+3OyVayO−2GFe+3:Fe+3:Va:O−2spinel + yFe+2TyCa+2OyVayO−2GFe+2:Ca+2:Va:O−2spinel+yFe+3TyCa+2OyVayO−2GFe+3:Ca+2:Va:O−2spinel + yAl+3TyVaOyVayO−2GAl+3:Va:Va:O−2spinel + yFe+2TyVaOyVayO−2GFe+2:Va:Va:O−2spinel + yFe+3TyVaOyVayO−2GFe+3:Va:Va:O−2spinel + yAl+3TyAl+3OyFe+2yO−2GAl+3:Al+3:Fe+2:O−2spinel + yAl+3TyVaOyFe+2yO−2GAl+3:Va:Fe+2:O−2spinel + yAl+3TyFe+2OyFe+2yO−2GAl+3:Fe+2:Fe+2:O−2spinel + yAl+3TyFe+3OyFe+2yO−2GAl+3:Fe+3:Fe+2:O−2spinel + yAl+3TyCa+2OyFe+2yO−2GAl+3:Ca+2:Fe+2:O−2spinel + yFe+2TyAl+3OyFe+2yO−2GFe+2:Al+3:Fe+2:O−2spinel + yFe+3TyAl+3OyFe+2yO−2GFe+3:Al+3:Fe+2:O−2spinel + yFe+2TyCa+2OyFe+2yO−2GFe+2:Ca+2:Fe+2:O−2spinel + yFe+3TyCa+2OyFe+2yO−2GFe+3:Ca+2:Fe+2:O−2spinel + yFe+2TyFe+2OyFe+2yO−2GFe+2:Fe+2:Fe+2:O−2spinel + yFe+3TyFe+2OyFe+2yO−2GFe+3:Fe+2:Fe+2:O−2spinel + yFe+2TyFe+3OyFe+2yO−2GFe+2:Fe+3:Fe+2:O−2spinel + yFe+3TyFe+3OyFe+2yO−2GFe+3:Fe+3:Fe+2:O−2spinel + yFe+2TyVaOyFe+2yO−2GFe+2:Va:Fe+2:O−2spinel + yFe+3TyVaOyFe+2yO−2GFe+3:Va:Fe+2:O−2spinel + RT(yAl+3TlnyAl+3T + yFe+2TlnyFe+2T+yFe+3TlnyFe+3T)+ 2RT(yAl+3OlnyAl+3O + yCa+2OlnyCa+2O+yFe+2OlnyFe+2O + yFe+3OlnyFe+3O+yVaOlnyVaO) + RT(yFe+2lnyFe+2 + yValnyVa + yFe+3TlnyFe+3T)  + 4RT(yO−2lnyO−2) + yAl+3TyFe+2TyAl+3OyVayO−2L 0Al+3,Fe+2:Al+3:Va:O−2spinel+ yAl+3TyAl+3OyFe+2OyVayO−2L 0Al+3:Al+3,Fe+2:Va:O−2spinel+ yAl+3TyFe+2TyFe+2OyVayO−2L 0Al+3,Fe+2:Fe+2:Va:O−2spinel+ yFe+2TyAl+3OyFe+2OyVayO−2L 0Fe+2:Al+3,Fe+2:Va:O−2spinel+ yAl+3TyFe+2OyVaOyVayO−2L 0Al+3:Fe+2,Va:Va:O−2spinel + yAl+3TyFe+3OyVaOyVayO−2L 0Al+3:Fe+3,Va:Va:O−2spinel

*T = tetrahedral sublattice, O = octahedral sublattice

The intermediate compound in the CaO-Al_2_O_3_-Fe_2_O_3_ system is C_2_F_3_A. In this work, it was modeled using the sublattice CEF model (Ca^+2^)_2_(Fe^+3^)_6_(Al^+3^)_12_(O^−2^)_14_. The Gibbs energy expression of C_2_F_3_A is given as follows:

(10) G 0−HSER = a + bT + cTlnT + dT2 + eT−1,

where $H^{\mathrm{SER}}$ represents the standard molar enthalpy of the pure elements (Ca, Al, Fe, and O) in their Standard Element Reference (SER) states at 298.15 K and 101,325 Pa (J·mol^−1^); T is the absolute temperature in Kelvin (K); and *a–e* are adjustable parameters to be optimized. 

Due to the lack of thermodynamic information for C(A,F)_3_, it was treated as a linear solid solution in this work. The sublattice CEF model is (Ca^+2^)_1_(Al^+3^, Fe^+3^)_6_(O^−2^)_10_. The Gibbs energy expression is given as follows:

(11) $$G_{m}^{{\mathrm{CN}}_{3}}\;-{\;H}^{\mathrm{SER}}\;={\;y}_{{\mathrm{Ca}}^{+2}}^{\prime }y_{{\mathrm{Al}}^{+3}}^{\prime \prime }y_{O^{-2}}^{\prime \prime \prime }G_{{\mathrm{Ca}}^{+2}:{\mathrm{Al}}^{+3}:O^{-2}}^{{\mathrm{CN}}_{3}}\;+{\;y}_{{\mathrm{Ca}}^{+2}}^{\prime }y_{{\mathrm{Fe}}^{+3}}^{\prime \prime }y_{O^{-2}}^{\prime \prime \prime }G_{{\mathrm{Ca}}^{+2}:{\mathrm{Fe}}^{+3}:O^{-2}}^{{\mathrm{CN}}_{3}}\;+\;\mathrm{RT}(y_{{\mathrm{Ca}}^{+2}}^{\prime }\ln y_{{\mathrm{Ca}}^{+2}}^{\prime })\;+\;6\mathrm{RT}(y_{{\mathrm{Al}}^{+3}}^{\prime \prime }\ln y_{{\mathrm{Al}}^{+3}}^{\prime \prime }+y_{{\mathrm{Fe}}^{+3}}^{\prime \prime }\ln y_{{\mathrm{Fe}}^{+3}}^{\prime \prime })\;+\;10\mathrm{RT}(y_{O^{-2}}^{\prime \prime \prime }\ln y_{O^{-2}}^{\prime \prime \prime })\;+{\;y}_{{\mathrm{Ca}}^{+2}}^{\prime }y_{{\mathrm{Al}}^{+3}}^{\prime \prime }y_{{\mathrm{Fe}}^{+3}}^{\prime \prime }y_{O^{-2}}^{\prime \prime \prime }L_{{\mathrm{Ca}}^{+1}:{\mathrm{Al}}^{+3},{\mathrm{Fe}}^{+3}:O^{-2}},$$

where $y^{\prime }$, $y^{\prime \prime }$, and $y^{\prime \prime \prime }$ represent the site fractions in the first, second, and third sublattices, respectively. $G_{m}^{{\mathrm{CN}}_{3}}$ denotes the Gibbs energy of CA_3_ and CF_3_. $L_{{\mathrm{Ca}}^{+1}:{\mathrm{Al}}^{+3},{\mathrm{Fe}}^{+3}:O^{-2}}\;$ is the interaction parameter optimized in the present work.

The halite phase was modeled using the sublattice CEF model (Ca^+2^, Fe^+2^, Fe^+3^, Va)_1_(O^−2^)_1_ with reference to the work of Gao et al. [65]. The Gibbs energy is expressed as follows:

(12) Gmhalite − HSER = yCa+2(1)yO−2(2)GCa+2:O−2halite + yFe+2(1)yO−2(2)GFe+2:O−2halite + yFe+3(1)yO−2(2)GFe+3:O−2halite + yVa(1)yO−2(2)GVa:O−2halite + RT(yCa+2(1)lnyCa+2(1) + yFe+2(1)lnyFe+2(1) + yFe+3(1)lnyFe+3(1) + yVa(1)lnyVa(1))+ RT(yO−2(2)lnyO−2(2)) + yCa+2(1)yFe+2(1)yO−2(2)LCa+2,Fe+2:O−2 0 + yCa+2(1)yFe+3(1)yO−2(2)LCa+2,Fe+3:O−2 0+ yFe+2(1)yFe+3(1)yO−2(2)LFe+2,Fe+3:O−2 0,

where $y^{\left(1\right)}$ and $y^{\left(2\right)}$ represent the site fractions in the first and second sublattices, respectively. $G_{m}^{\mathrm{halite}}\;$ denotes the Gibbs energy of Halite.

Significant solubility of Al_2_O_3_ and Fe_2_O_3_ is present in both C_2_F and calcium aluminate (C_3_A, CA, CA_2_, and CA_6_) phases, arising primarily from the mutual substitution between Al^+3^ and Fe^+3^ [60,61,63,75]. Therefore, in the CaO-Al_2_O_3_-Fe_2_O_3_ system, C_3_AF, CAF, CAF_2_, CAF_6_, and C_2_AF were treated as solid solutions in this work. They were described using the sublattice CEF model (Ca^+2^)_x_(Al^+3^, Fe^+3^)_y_(O^−2^)_z_. Taking CAF_2_ as an example, its Gibbs energy expression is given as follows:

(13) $$G_{m}^{{\mathrm{CAF}}_{2}}\;-\;H^{\mathrm{SER}}\;={\;y}_{{\mathrm{Ca}}^{+2}}^{a}y_{{\mathrm{Al}}^{+3}}^{b}y_{O^{-2}}^{c}G_{{\mathrm{Ca}}^{+2}:{\mathrm{Al}}^{+3}:O^{-2}}^{{\mathrm{CAF}}_{2}}\;+\;y_{{\mathrm{Ca}}^{+2}}^{a}y_{{\mathrm{Fe}}^{+3}}^{b}y_{O^{-2}}^{c}G_{{\mathrm{Ca}}^{+2}:{\mathrm{Fe}}^{+3}:O^{-2}}^{{\mathrm{CAF}}_{2}}\;+\;\mathrm{RT}(y_{{\mathrm{Ca}}^{+2}}^{a}\ln y_{{\mathrm{Ca}}^{+2}}^{a})\;+\;4\mathrm{RT}(y_{{\mathrm{Al}}^{+3}}^{b}\ln y_{{\mathrm{Al}}^{+3}}^{b}\;+\;y_{{\mathrm{Fe}}^{+3}}^{b}\ln y_{{\mathrm{Fe}}^{+3}}^{b})\;+\;7\mathrm{RT}(y_{O^{-2}}^{c}\ln y_{O^{-2}}^{c})\;+{\;y}_{{\mathrm{Ca}}^{+2}}^{a}y_{{\mathrm{Al}}^{+3}}^{b}y_{{\mathrm{Fe}}^{+3}}^{b}y_{O^{-2}}^{c}L_{{\mathrm{Ca}}^{+1}:{\mathrm{Al}}^{+3},{\mathrm{Fe}}^{+3}:O^{-2}},$$

where $y^{a}$, $y^{b}$, and $y^{c}$ represent the site fractions in the first, second, and third sublattices, respectively. $G_{m}^{{\mathrm{CAF}}_{2}}$ denotes the Gibbs energy of CA_2_ and CF_2_. $L_{{\mathrm{Ca}}^{+1}:{\mathrm{Al}}^{+3},{\mathrm{Fe}}^{+3}:O^{-2}}$ is the interaction parameter optimized in this work.

## 4. Thermodynamic Calculations

In the present work, the CaO-Fe_2_O_3_ and the CaO-Al_2_O_3_-Fe_2_O_3_ system were optimized based on experimental data taken from the literature using the PARROT module in the Thermo-Calc software N Version [76], which utilizes the least-squares method to minimize the sum of squared differences between calculated and experimental data. Eventually, a self-consistent set of thermodynamic parameters was obtained that effectively describes the phase equilibria and thermodynamic properties of the CaO-Al_2_O_3_-Fe_2_O_3_ system, as presented in Table 2.

### 4.1. CaO-Fe_2_O_3_ System

The thermodynamic parameters of the intermediate compounds CF and C_2_F were optimized using the experimental enthalpy data reported by Bonnickson [39], whereas those of CF_2_ were assessed based on the optimized results reported by Selleby [43]. For the liquid phase of the CaO-Fe_2_O_3_ system, the interaction parameters $L_{{\mathrm{Ca}}^{+2},{\mathrm{Fe}}^{+2}:O^{-2}}^{\mathrm{liquid}}$, $L_{{\mathrm{Ca}}^{+2}:O^{-2},{\mathrm{FeO}}_{1.5}}^{\mathrm{liquid}}$*, and* $L_{{\mathrm{Ca}}^{+2},{\mathrm{Fe}}^{+2}:O^{-2},{\mathrm{FeO}}_{1.5}}^{\mathrm{liquid}}$ were introduced during the optimization. The optimization of the liquid phase was primarily based on the liquidus data reported by Cheng et al. [33].

**Figure 3:**
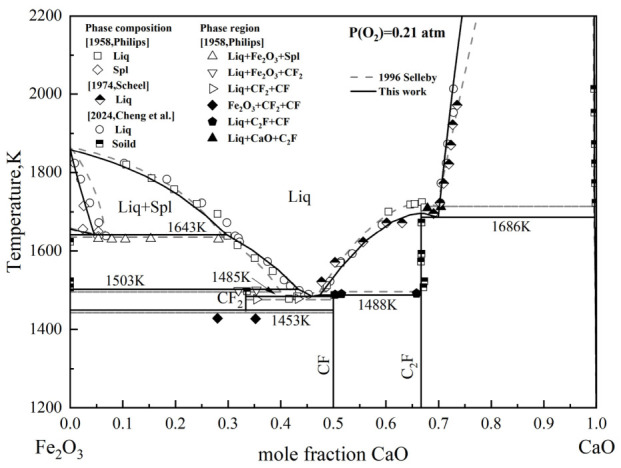
Calculated phase diagram of the CaO-Fe_2_O_3_ binary system at P(O_2_) = 0.21 atm compared with the experimental data [30,31,33,43].

Figure 3 shows the calculated phase diagram of the CaO-Fe_2_O_3_ system obtained in the present work. The gray dashed lines represent the thermodynamic description reported by Selleby [43], in which the liquid phase was modeled using the ionic two-sublattice model. In contrast, the optimized results obtained in the present work reproduce the liquidus reported by Cheng et al. [33] more accurately, resulting in a more reasonable representation of the experimental data for the CaO-Fe_2_O_3_ system.

**Figure 4:**
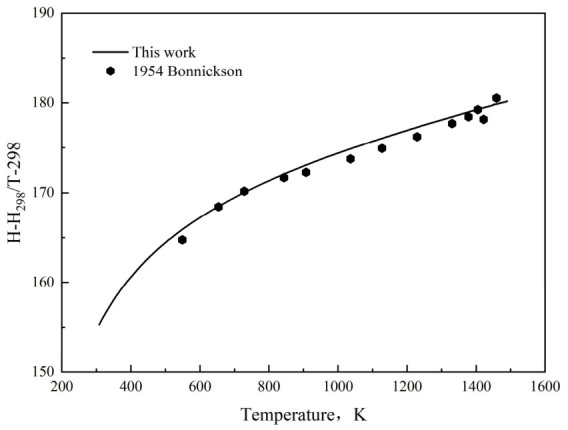
Calculated heat content of CF compared with the experimental data [39].

**Figure 5:**
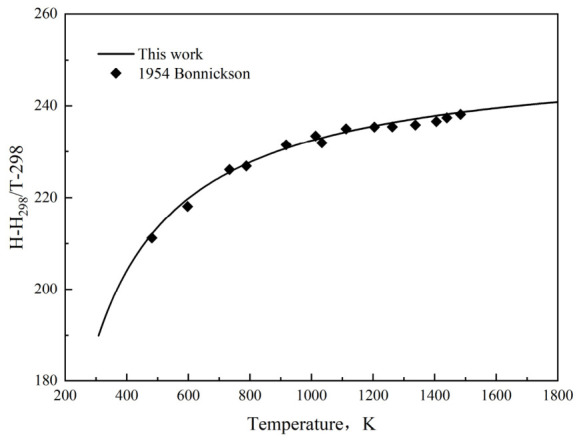
Calculated heat content of C_2_F compared with the experimental data [39].

Figure 4 shows the calculated enthalpy of CF. The calculated results are in good agreement with the experimental measurements reported by Bonnickson [39]. As shown in Figure 5, the calculated enthalpy of C_2_F also exhibits excellent agreement with the experimental data [39].

Table 3 summarizes the comparison between the experimentally determined invariant reactions and the calculated results. The calculated temperature of the eutectic reaction, Liquid ↔ CF_2_ + CF, is 1485 K, which agrees well with the experimental value of 1488 K reported by Cheng et al. [33]. The calculated temperature of the peritectic reaction, Fe_2_O_3_ + Liquid ↔ C_2_F, is 1503 K, showing excellent agreement with the experimental value of 1508 K. Overall, the optimized thermodynamic parameters accurately reproduce the liquidus data reported by Cheng et al. [33].

### 4.2. CaO-Al_2_O_3_-Fe_2_O_3_ System

For the CaO-Al_2_O_3_-Fe_2_O_3_ system, the solid-solution phases C(A,F)_3_, C_3_AF, CAF, CAF_2_, CAF_6_, and C_2_AF were considered. To reproduce the experimentally observed phase-equilibrium data, including both two-phase and three-phase regions, the parameters GCa+2:Al+3:O−2C(A,F)3 0, GCa+2:Fe+3:O−2C(A,F)3 0, LCa+2:Al+3,Fe+3:O−2C(A,F)3 0, GCa+2:Fe+3:O−2C3AF 0,LCa+2:Al+3,Fe+3:O−2C3AF 0, LCa+2:Al+3,Fe+3:O−2CAF 0, LCa+2:Al+3,Fe+3:O−2CAF2 0, GCa+2:Fe+3:O−2CAF6 0,LCa+2:Al+3,Fe+3:O−2CAF6 0, GCa+2:Al+3:O−2C2AF 0, and LCa+2:Al+3,Fe+3:O−2C2AF 0 were introduced and optimized. In particular, the Gibbs energy of the hypothetical end member C_3_F was optimized to reproduce the experimentally measured Fe_2_O_3_ solubility in C_3_A reported by Hassaan et al. [64], whereas the Gibbs energies of the hypothetical end members CF_6_ and C_2_A, together with their corresponding interaction parameters, were constrained by the phase-equilibrium data reported by Cheng et al. [62]. To reproduce the phase equilibria involving C_2_F_3_A, including both two-phase and three-phase regions, the parameter GCa+2:Fe+3:Al+3:O−2C2F3A 0 was introduced and optimized. The thermodynamic optimization of the liquid phase in the CaO-Al_2_O_3_-Fe_2_O_3_ system was primarily based on the phase-equilibrium data reported by Cheng et al. [62]. During the optimization, the interaction parameter $L_{{\mathrm{Ca}}^{+2}:{\mathrm{AlO}}_{1.5},{\mathrm{FeO}}_{1.5},O^{-2}}^{\mathrm{liquid}}$ was introduced.

**Figure 6:**
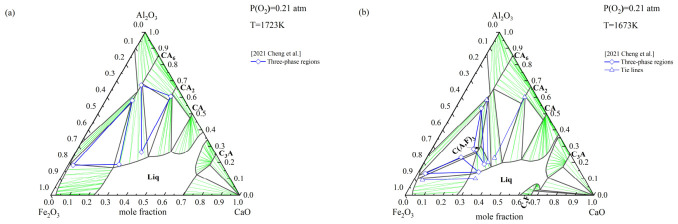

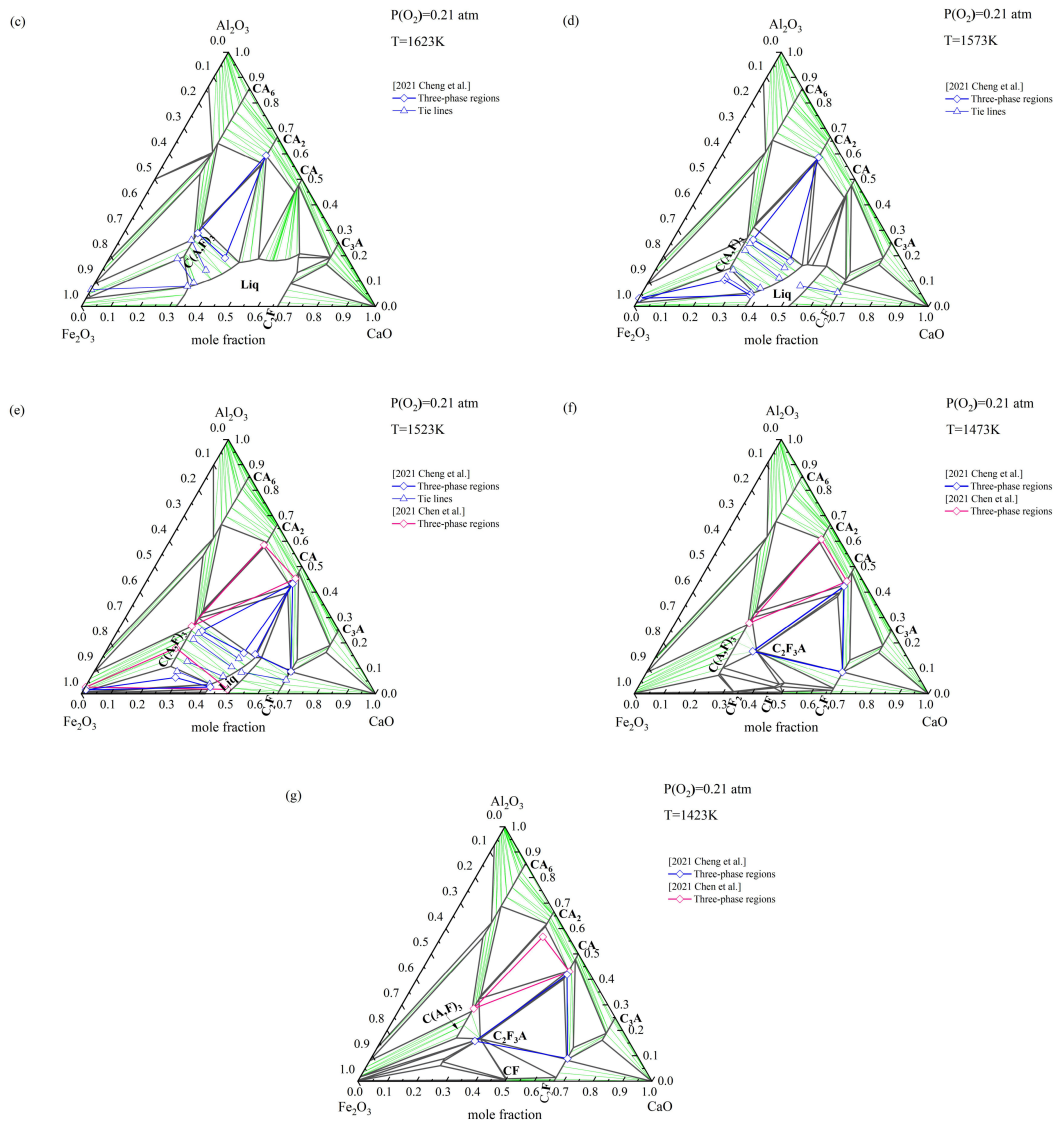
Calculated isothermal sections of the CaO-Al_2_O_3_-Fe_2_O_3_ system at P(O_2_) = 0.21 atm with the experimental data [62,63] (**a**) 1723 K; (**b**) 1673 K; (**c**) 1623 K; (**d**) 1573 K; (**e**) 1523 K; (**f**) 1473 K; (**g**) 1423 K.

Figure 6a–g show the calculated isothermal sections at 1723, 1673, 1623, 1573, 1523, 1473, and 1428 K, respectively, together with the experimental data [62,63]. As shown in Figure 6a–g, the calculated liquidus boundaries, two-phase regions, and three-phase regions are in good agreement with the experimental observations, demonstrating the reliability of the present thermodynamic description of the CaO-Al_2_O_3_-Fe_2_O_3_ system.

**Figure 7:**
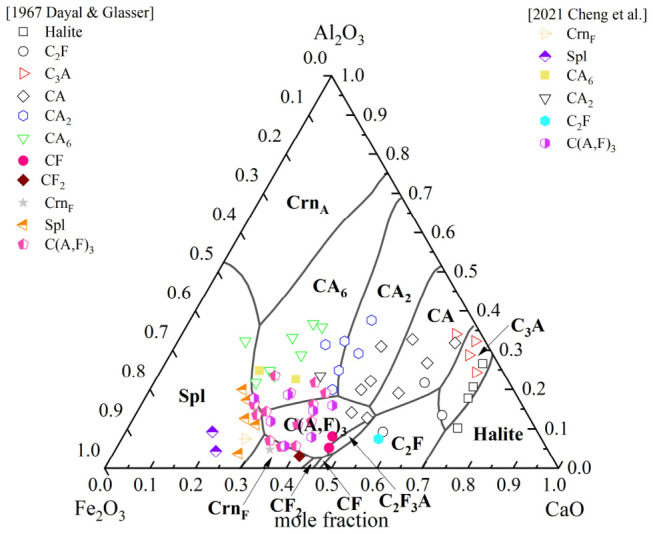
Calculated primary phase regions of the CaO-Al_2_O_3_-Fe_2_O_3_ system [60,62].

Figure 7 shows the calculated primary crystallization phase fields of the CaO-Al_2_O_3_-Fe_2_O_3_ ternary system. A total of thirteen primary phase fields are predicted, including CA_6_, CA_2_, CA, C_3_A, C_2_F, CF, CF_2_, C(A,F)_3_, C_2_F_3_A, Crn_A_, Crn_F_, Spl, and Halite.

**Figure 8:**
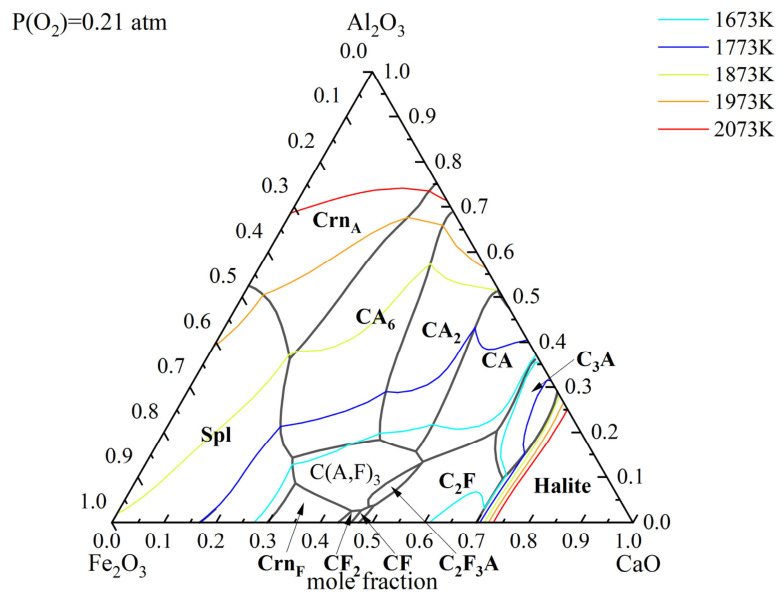
Calculated liquidus projection of the CaO-Al_2_O_3_-Fe_2_O_3_ system.

The calculated liquidus projection of the CaO-Al_2_O_3_-Fe_2_O_3_ system is presented in Figure 8. Table 4 compares the calculated invariant reactions with the available literature data. Overall, the calculated temperatures and phase compositions show satisfactory agreement with the reported values. Nevertheless, discrepancies remain for some invariant reactions, which are estimated in the literature rather than directly determined experimentally and therefore involve greater uncertainty. Despite these local deviations, the present thermodynamic description provides a consistent representation of the overall phase equilibria in the CaO-Al_2_O_3_-Fe_2_O_3_ system.

## 5. Conclusions

The CaO-Fe_2_O_3_ binary system and the CaO-Al_2_O_3_-Fe_2_O_3_ ternary system were systematically assessed using the CALPHAD approach. The main conclusions are summarized as follows:

(1) The CaO-Fe_2_O_3_ binary system was re-optimized using the ionic two-sublattice model, and a self-consistent set of thermodynamic parameters was established. The calculated results were in good agreement with the available phase-diagram data and thermodynamic properties of the intermediate compounds, demonstrating the reliability of the present thermodynamic description.

(2) Based on the thermodynamic parameters of the constituent binary systems and the available experimental data from the literature, the CaO-Al_2_O_3_-Fe_2_O_3_ ternary system was thermodynamically optimized using the CALPHAD method and the ionic two-sublattice model. The resulting thermodynamic description showed good agreement with the available experimental data and was able to satisfactorily reproduce the phase equilibria and thermodynamic properties of the system within experimental uncertainties.

(3) The thermodynamic assessment of the CaO-Al_2_O_3_-Fe_2_O_3_ system conducted in this study is crucial for controlling phase transformations during material synthesis and application.

## Figures and Tables

**Table 1:** Thermodynamic models of all stable phases in the CaO-Al_2_O_3_-Fe_2_O_3_ system.

Phase	Crystal System	Space Group	Phase Prototype	Model	Notation Used in This Study	Ref.
liquid	—	—	—	(Al^+3^, Fe^+2^, Ca^+2^) _P_ (O^−2^, AlO_1.5_, FeO_1.5_, Va, O) _Q_	Liq	—
Corundum	Trigonal	R$\bar{3}c$	Al_2_O_3_	(Al^+3^, Fe^+3^)_2_(O^−2^)_3_	Crn, Al_2_O_3_(Crn_A_), Fe_2_O_3_(Crn_F_)	[66]
Fe_2_Al_2_O_6_	Orthorhombic	Pna2_1	FeAlO_3_	(Al^+3^)_2_(Fe^+3^)_2_(O^−2^)_6_	Fe_2_Al_2_O_6_	[59]
Spinel	Cubic	Fd$\bar{3}$m1	MgAl_2_O_4_	(Al^+3^, Fe^+2^, Fe^+3^)_1_ (Al^+3^, Ca^+2^, Fe^+2^, Fe^+3^, Va)_2_ (Fe^+2^, Va)_2_(O^−2^)_4_	Spl	[59]
Halite	Cubic	Fm$\bar{3}$m	NaCl	(Ca^+2^, Fe^+2^, Fe^+3^,Va)_1_(O^−2^)_1_	Halite	[67]
C_2_F_3_A	Orthorhombic	Pnma	—	(Ca^+2^)_2_(Fe^+3^)_6_(Al^+3^)_2_(O^−2^)_14_	C_2_F_3_A	[68]
Ca(Al,Fe)_6_O_10_	Hexagonal	P6_3_/mmc	CaAl_6_O_10_	(Ca^+2^)_1_(Al^+3^, Fe^+3^)_6_(O^−2^)_10_	C(A,F)_3_	[69]
C_3_AF	Cubic	Ia	Ca_3_Al_2_O_6_	(Ca^+2^)_3_(Al^+3^, Fe^+3^)_2_(O^−2^)_6_	C_3_A, C_3_AF	[70]
CAF	Monoclinic	P2_1_/c	CaAl_2_O_4_	(Ca^+2^)_1_(Al^+3^, Fe^+3^)_2_(O^−2^)_4_	CA, CF, CAF	[71]
CAF_2_	Monoclinic	C2/c	CaAl_4_O_7_	(Ca^+2^)_1_(Al^+3^, Fe^+3^)_4_(O^−2^)_7_	CA_2_, CF_2_, CAF_2_	[72]
CAF_6_	Hexagonal	P6_3_/mmc	BaFe_12_O_19_	(Ca^+2^)_1_(Al^+3^, Fe^+3^)_12_(O^−2^)_19_	CA_6_, CAF_6_	[73]
C_2_AF	Hexagonal	Pnma	Ca_2_Fe_2_O_5_	(Ca^+2^)_2_(Al^+3^, Fe^+3^)_2_(O^−2^)_5_	C_2_F, C_2_AF	[74]

**Table 2:** Thermodynamic model and optimization results of thermodynamic parameters for the CaO-Al_2_O_3_-Fe_2_O_3_ ternary system *.

Phase	Thermodynamic Model	Thermodynamic Parameter
liquid	(Al^+3^, Fe^+2^, Ca^+2^) _P_ (O^−2^, AlO_1.5_, FeO_1.5_, Va, O) _Q_	LCa+2,Fe+2:O−2liquid=−121000+2T 0
		LCa+2,Fe+2:O−2liquid=−40000+20T 1
		LCa+2,Fe+2:O−2liquid=+2400 2
		LCa+2:O−2,FeO1.5liquid 0=−238000+12T
		LCa+2:O−2,FeO1.5liquid 1=−55300
		LCa+2:O−2,FeO1.5liquid 2=+144000
		LCa+2,Fe+2:O−2,FeO1.5liquid 0=+65700
		LCa+2:O−2,AlO1.5,FeO1.5liquid 0=−420598.4120T
		LCa+2:O−2,AlO1.5,FeO1.5liquid 1=+306238.41+60T
		LCa+2:O−2,AlO1.5,FeO1.5liquid 2=−20698.41−40T
C_2_F_3_A	(Ca^+2^) _2_ (Fe^+3^) _6_ (Al^+3^) _2_ (O^−2^) _14_	GCa+2:Fe+3:Al+3:O−2C2F3A 0=−5922501+4178.013T−659.886Tln(T)+3.89544 × 10−4T2 +7838507.05*T*^−1^
C(A,F)_3_	(Ca^+2^) _1_ (Al^+3^, Fe^+3^) _6_ (O^−2^) _10_	GCa+2:Al+3:O−2C(A,F)3 0=+GCaOS+3GAl2O3S−22531.16+40T
		GCa+2:Fe+3:O−2C(A,F)3 0=+GCaOS+3GFe2O3S−48577.7+12.52T
		LCa+2:Al+3,Fe+3:O−2C(A,F)3 0=−56000 − 81T
C_3_AF	(Ca^+2^) _3_ (Al^+3^, Fe^+3^) _2_ (O^−2^) _6_	GCa+2:Fe+3:O−2C3AF 0=+3GCaOS+GFe2O3S − 20000
		LCa+2:Al+3,Fe+3:O−2C3AF 0=−105198.708+12.698T
CAF	(Ca^+2^) _1_ (Al^+3^, Fe^+3^) _2_ (O^−2^) _4_	GCa+2:Fe+3:O−2CAF 0=−1536066+1010.683T − 170.6283Tln(T) −0.007661T2+916880T−1
		LCa+2:Al+3,Fe+3:O−2CAF 0=+23255+9T
CAF_2_	(Ca^+2^) _1_ (Al^+3^, Fe^+3^) _4_ (O^−2^) _7_	GCa+2:Fe+3:O−2CAF2 0=+GCaOS+2GFe2O3S+22358 − 41.337T
		LCa+2:Al+3,Fe+3:O−2CAF2 0=+140520 − 38.5T
CAF_6_	(Ca^+2^) _1_ (Al^+3^, Fe^+3^) _12_ (O^−2^) _19_	GCa+2:Fe+3:O−2CAF6 0=+GCaOS+6GFe2O3S+60000
		LCa+2:Al+3,Fe+3:O−2CAF6 0=+60000 − 40T
C_2_AF	(Ca^+2^) _2_ (Al^+3^, Fe^+3^) _2_ (O^−2^) _5_	GCa+2:Al+3:O−2C2AF 0=+2GCaOS+GAl2O3S − 20000
		GCa+2:Fe+3:O−2C2AF 0=−2227283+1525.422T −251.3585Tln(T)+2819950T−1
		LCa+2:Al+3,Fe+3:O−2C2AF 0=+20040 − 50T

* Only the parameters optimized in the present work are listed. The thermodynamic parameters for the CaO-Al_2_O_3_ and Al_2_O_3_-Fe_2_O_3_ systems were adopted from the assessments reported by Ye et al. [29] and Dreval et al. [59], respectively.

**Table 3:** Comparison of calculated and experimental results of invariant equilibrium reaction of CaO-Fe_2_O_3_ binary system.

Equilibrium	Temperature (K)	Composition of Liquid/mol.%		Ref.
		CaO	Fe_2_O_3_	
Liquid ↔ CF_2_ + CF	1488	55.0	45.0	[33]-Exp *
	1476	58.0	42.0	[43]-Cal *
	1485	55.0	45.0	TW *
Liquid ↔ C_2_F + CaO	1713	33.0	67.0	[43]-Cal
	1686	30.0	70.0	TW
Liquid + Fe_2_O_3_ ↔ C_2_F	1508	58.0	42.0	[33]-Exp
	1496	53.0	47.0	[43]-Cal
	1503	57.0	43.0	TW
Liquid + C_2_F ↔ CF	1496	53.0	47.0	[43]-Cal
	1488	53.0	47.0	TW

* Note: Exp means Experiment; Cal means Calculation; TW means This Work.

**Table 4:** Comparison of calculated and experimental results of invariant equilibrium reaction of CaO-Al_2_O_3_-Fe_2_O_3_ ternary system.

Type	Equilibrium	Temperature (K)	Composition of Liquid/mol.%			Ref.
			CaO	Al_2_O_3_	Fe_2_O_3_	
Peritectic (P_1_)	Liquid + Spl + CA_6_ ↔ C(A,F)_3_	1686	31.0	17.0	52.0	[62]-Exp *
		1684	29.0	15.0	56.0	[65]-Cal *
		1686	28.0	14.0	58.0	TW *
Peritectic (P_2_)	Liquid + Spl + C(A,F)_3_ ↔ Crn_F_	1643	32.0	10.0	58.0	[62]-Exp
		1638	31.0	8.0	61.0	[65]-Cal
		1642	30.0	9.0	61.0	TW
Eutectic (E_1_)	Liquid ↔ C_2_F + CF + C_2_F_3_A	1457	47.0	5.0	48.0	[65]-Cal
		1470	48.0	4.0	48.0	TW
Transition (U_1_)	Liquid + CaO ↔ C_2_F + C_3_A	1655	71.0	14.0	15.0	[62]-Est
		1638	70.0	17.0	13.0	[65]-Cal
		1658	70.0	10.0	20.0	TW
Transition (U_2_)	Liquid + C_3_A ↔ C_2_F + CA	1597	66.0	29.0	5.0	[62]-Est
		1575	64.0	31.0	5.0	[65]-Cal
		1618	64.0	20.0	16.0	TW
Transition (U_3_)	Liquid + C(A,F)_3_ ↔ CF + C_2_F_3_A	1458	46.0	4.0	50.0	[65]-Cal
		1473	47.0	4.0	49.0	TW
Transition (U_4_)	Liquid + CA_2_ ↔ CA + C(A,F)_3_	1533	46.0	17.0	37.0	[62]-Exp
		1572	47.0	20.0	33.0	[65]-Cal
		1553	50.0	16.0	34.0	TW
Transition (U_5_)	Liquid + CA_6_ ↔ CA_2_ + C(A,F)_3_	1648	37.0	19.0	44.0	[62]-Exp
		1649	41.0	22.0	37.0	[65]-Cal
		1648	42.0	18.0	40.0	TW
Transition (U_6_)	Liquid + Crn_A_ ↔ CA_6_ + Spl	1934	5.0	41.0	55	[62]-Est
		1893	17.0	43.0	40.0	[65]-Cal
		1864	16.0	36.0	48.0	TW
Transition (U_7_)	Liquid + Crn_F_ ↔ CF_2_ + C(A,F)_3_	1498	43.0	2.0	55.0	[62]-Exp
		1483	41.0	1.0	58.0	[65]-Cal
		1483	44.0	3.0	53.0	TW
Transition (U_8_)	Liquid + CF_2_ ↔ CF + C(A,F)_3_	1467	45.0	2.0	53.0	[62]-Exp
		1465	43.0	1.0	56.0	[65]-Cal
		1472	47.0	3.0	50.0	TW

* Note: Exp means Experiment; Cal means Calculation; Est means Estimated; TW means This Work.

## Data Availability

The original contributions presented in the study are included in the article; further inquiries can be directed to the corresponding authors.
